# Potential of *in vivo* real-time gastric gas profiling: a pilot evaluation of heat-stress and modulating dietary cinnamon effect in an animal model

**DOI:** 10.1038/srep33387

**Published:** 2016-09-16

**Authors:** Jian Zhen Ou, Jeremy J. Cottrell, Nam Ha, Naresh Pillai, Chu K. Yao, Kyle J. Berean, Stephanie A. Ward, Danilla Grando, Jane G. Muir, Christopher J. Harrison, Udani Wijesiriwardana, Frank R. Dunshea, Peter R. Gibson, Kourosh Kalantar-zadeh

**Affiliations:** 1 School of Engineering, RMIT University, Melbourne, Australia; 2 Faculty of Veterinary and Agricultural Sciences, The University of Melbourne, Parkville, Australia; 3 Department of Gastroenterology, The Alfred Hospital, Monash University, Melbourne, Australia; 4 Monash Ageing Research Centre, Monash University, Melbourne, Australia; 5 School of Science, RMIT University, Bundoora, Australia

## Abstract

Gastroenterologists are still unable to differentiate between some of the most ordinary disorders of the gut and consequently patients are misdiagnosed. We have developed a swallowable gas sensor capsule for addressing this. The gases of the gut are the by-product of the fermentation processes during digestion, affected by the gut state and can consequently provide the needed information regarding the health of the gut. Here we present the first study on gas sensor capsules for revealing the effect of a medical supplement in an animal (pig) model. We characterise the real-time alterations of gastric-gas in response to environmental heat-stress and dietary cinnamon and use the gas profiles for understanding the bio-physiological changes. Under no heat-stress, feeding increases gastric CO_2_ concentration, while dietary cinnamon reduces it due to decrease in gastric acid and pepsin secretion. Alternatively, heat-stress leads to hyperventilation in pigs, which reduces CO_2_ concentration and with the cinnamon treatment, CO_2_ diminishes even more, resulting in health improvement outcomes. Overall, a good repeatability in gas profiles is also observed. The model demonstrates the strong potential of real-time gas profiler in providing new physiological information that will impact understanding of therapeutics, presenting a highly reliable device for monitoring/diagnostics of gastrointestinal disorders.

Currently, gastroenterology is still mired with patients’ problems stemming from misdiagnosis^1^,^2^,^3^,^4^. In many cases, due to the lack of direct physiological evidences, gastroenterologists can only rely on physical symptoms rather than associated hard data to diagnose the diseases and disorders of the gastrointestinal tract^5^,^6^. Conventional medical devices including acoustic and electromagnetic based systems and endoscopy are only able to provide diagnostic imaging on limited types of gastrointestinal disorders such as ulcer, tumour related illnesses and inflammable bowel diseases (IBD)^7^,^8^. However, diagnostic imaging is ineffective for many other very frequent disorders of the gut such as irritable bowel syndromes (IBS)^6^.

It is now widely accepted that gastrointestinal gas profiles provide key information regarding the gut’s state-of-health^9^,^10^. Based on indirect measurements using commonly implemented breath analysis and direct measurements dates back to 1980s or earlier, the concentration and types of gases produced in the gut are affected by gastrointestinal tract physiological functions, and may be involved in the pathogenesis of some gastrointestinal tract disorders^9^,^10^. Gases presented in the gut, commonly H_2_, CH_4_, CO_2_, NO_x_ and H_2_S as well as many volatile organic compounds, are mainly the result of the fermentation processes and other activities by the intestinal microbial community and may appear in the metabolic pathways^9^,^10^. So far, many *in vivo* and *in vitro* studies have shown that there are strong links between the intestinal gases and different types of gastrointestinal disorders. For instance, H_2_ and CH_4_ are used for detecting and characterising carbohydrate malabsorption^11^. CH_4_ has been found to play an important role in the peristaltic control of the gut and its presence is closely correlated to constipation-type IBS^10^,^12^. H_2_S and NO_x_ have been identified as gasotransmitters that are important for the intestinal motility and secretory functions. Both of them are also considered as typical markers for IBD^13^,^14^,^15^,^16^.

Nevertheless, the accuracies and reliabilities of the current intestinal gas measurements have been of great concerned. Breath analysis is currently the most common indirect method which relies on the measurement of intestinal gases that are first absorbed onto the gut walls, then recirculated to the lung through the blood stream and eventually excreted by respiration^11^,^17^,^18^. Misleading diagnostic information may be given due to interfering gases produced by the interfering parts of the human body and activities which are highly dependent on the metabolic and physiological nature of individuals and also environmental effects^9^,^19^. Another common indirect approach is to measure the headspace gas produced from *in vitro* fermentation of faecal samples^20^,^21^,^22^,^23^. This method results in more accurate assessment outcomes but limits to the information of the microbial community of the distal colon area. As such, it is critical to measure intestinal gases directly from where they are produced in the gastrointestinal tract for providing more accurate information regarding the physiological abnormities of the gut.

Early attempt of direct and accurate intestinal gas measurements have been realised by inserting tubes into patients’ oral cavity or anus to collect gas samples^24^,^25^. However, such approaches are no longer applied in nowaday gastroenterology community due to their invasive and inconvenient nature.

Recently, we have successfully developed a non-invasive and direct gas profiling tool called “gas sensor capsule” (Fig. 1). The capsule is swallowable but indigestible, which has enabled real-time and precise gas measurements in different sections of the gastrointestinal tract in animal models^26^. The emergence of such a novel tool can therefore potentially provide a new approach *in vivo* for the investigation of gut under various disease and disorder conditions. The gas capsule can also be a unique tool in assessing therapeutics and their impacts on the gut. However, such capabilities have not been shown yet.

Here, such hypothesises are validated using an animal model influenced by a representative gut disorder and a dietary therapeutic agent. The model of gut disorder is induced by stressing pigs under cyclical heat. The heat-stress effects on the gut physiological abnormalities are well-known in the literature. When under heat-stress, the shunting of blood from the central circulation to the periphery reduces the perfusion to the viscera, particularly the gastrointestinal tract, which may result in tissue hypoxia and intestinal mucosal injury^27^. Damage to the integrity of the intestinal wall has been linked to a reduction in permeability and associated with gut inflammation and endotoxemia^28^,^29^, which can significantly alter nutrient digestive and absorptive functionalities and hence the intestinal gas profile^27^,^30^,^31^. On the other hand, supplemental cinnamon (*cinnamomum verum*) is chosen as a representative therapeutic agent due to its anti-inflammatory, anti-oxidant and free radical scavenging properties in the gastrointestinal tract^32^,^33^. These advantageous characterises make this spice an ideal candidate for maintaining normal digestive functionalities and intestinal barrier integrity under the heat-stress condition, while at the same time it has relatively minor adverse-effects that cause minimum disturbance on the intestinal microbial community.

In this pilot study, the capability of gas sensor capsule is for the first time investigated through examining gastric gas production in pigs under heat-stress and assessing the effect of oral cinnamon on such gas profiles. The results are interpreted in the light of other complementary physiological and biochemical assessments. The repeatability of the gas profiles is also evaluated in a large group of pigs for revealing the effectiveness of this novel medical tool.

## Results

### Thermoneutral conditions

The real-time gastric CO_2_ gas profile of the thermoneutral control pig (no cinnamon in the diet) was measured by the gastric gas profiler (a modified version of gas sensor capsule with higher density) and shown in Fig. 2A. The first feeding of the day resulted in an increase of CO_2_ level by ~21% in ~3–4 h, while there was a ~8% increase of CO_2_ concentration at ~3 h after the second feeding. Simultaneously, a rise of gastric temperature of up to 0.8 °C corresponding to each feeding was observed. A rapid decline of CO_2_ gas level was seen after reaching the maximum of the day.

The gastric CO_2_ gas pattern and production kinetics in the thermoneutral cinnamon-treated pig (Fig. 2B) were distinctly different from that observed in the control pig. In particular, the CO_2_ concentration level increased only by ~13% and ~6% after the first and second feeding, respectively, which were markedly lower than that of thermoneutral control and were consistent with the decrease of the blood CO_2_ levels presented in Table 1. It was also noted that a decrease in CO_2_ concentration was seen immediately after the second feeding, which was associated with a ~50% reduction of the metabolic heat production (temperature fluctuation was less than 0.4 °C that was just above the noise floor of the temperature sensor).

Addition of cinnamon in the diet significantly increased the gastric pH from ~1 to ~3.2 (Fig. 3D), with the anticipated concomitant decrease in blood bicarbonate level (Table 1) as it is concurrently released together with gastric acid during the formation of the alkaline tide. Raman spectroscopy was used for assessing the pepsin level in the stomach of the thermoneutral pigs. In the Raman spectrum of gastric digesta, distinct peaks representing the unique markers of pepsin^34^,^35^ were observed (Fig. 4). In particular, the Raman peaks at ~1450, 1555, 1610 and 1675 cm^−1^ could be ascribed to the stretching modes of C-H, amide NH, C=C and amide CH bonds, respectively. In the pig treated with cinnamon, the normalised intensities of these Raman peaks were much lower than those of control, indicating less pepsin concentration in the gastric digesta.

### Effects of heat-stress

When under heat-stress, gastric pH increased (Fig. 3D) and the gastric CO_2_ pattern was changed compared to those of the thermoneutral control pig. As shown in Fig. 2C, a CO_2_ concentration maximum was observed at ~4 h after the first feeding for the heat-stress controlled pig (no cinnamon supplement) with the value comparable to that in the thermoneutral control animal. At the same time, a significant increase in the gastric temperature by ~3 °C was observed. However, the second feeding resulted in a very small increase in the CO_2_ concentration, which was only ~2% in comparison to ~8% in the thermoneutral condition. This was associated with a decrease of blood CO_2_ and carbonate concentrations compared to that under thermoneutral controlled condition, while their blood pH values were similar (Table 1).

During heat-stress, physiological indices of the pig including respiratory rate, skin and rectal temperatures were all increased, as expected from the literature (Fig. 3A–C)^36^,^37^. In particular, the respiration rate increased from ~30/min to ~140/min at 13:00 and reached a maximum of ~180/min at 15:00 (Fig. 3A), while the skin temperature plateaued much earlier at 13:00 (Fig. 3B). After maximal respiration rate, there was a rapid decrease in the gastric temperature from the peak of ~42 °C down to ~41 °C at the end of the heat-stress period (17:00).

Cinnamon ingestion under heat-stress conditions was associated with a considerably altered CO_2_ gas pattern (Fig. 2D). From Fig. 2D, after the first feeding with cinnamon supplement, the CO_2_ concentration was increased by ~17% which was 3% lower than that in the heat-stress control subject. The influence of cinnamon on the CO_2_ gas profile was more pronounced after 13:00. For the heat-stress cinnamon-treated subject, a rapid drop by 7% in the CO_2_ concentration was observed and the second feeding of the day did not result in the usual immediate increase in CO_2_ production as seen in Fig. 2C. Metabolic heat production corresponding to the feeding was also noticed (Fig. 2D). The abnormalities of pH (Fig. 3D) and pepsin levels (Fig. 4) were attenuated in the cinnamon-treated heat-stress pig compared with its corresponding control.

Likewise, the rise of gastric (Fig. 2D) and skin temperatures (Fig. 3B) due to heat-stress reduced from average ~3.3 °C to ~2.5 °C, and ~3.8 °C to ~3.2 °C, respectively, compared to those in the heat-stress control. Despite the conspicuous effect of the cinnamon supplement on the gastric indices, no distinct difference in rectal temperature profile was observed (Fig. 3C).

### Evaluation of the repeatability of gastric gas profiles

Figure 2 demonstrated that all timepoint measurements of CO_2_ were within a narrow band of variation within the overall curve of the graphs produced. Additionally the CO_2_ patterns were remarkably similar for Day 1 and 2 indicating the repeatability of gas profiles generated for all four test conditions. For both thermoneutral and heat-stress conditions of the control subjects, the overall CO_2_ concentrations across the first and second day showed less than 1% variance. There was a slight decrease of ~3% and ~5% on the second day for the cinnamon-treated subjects under thermoneutral and heat-stress, respectively.

Repeatability was further tested through the use of two additional pigs in each test group using normal-density gas sensor capsules that left the pigs’ bodies within a normal bowel transit time. The profile obtained by the normal-density capsules showed very strong consistency with those obtained by high-density gastric gas profilers that were immobilized in the pigs’ stomach (Fig. 5). As such, during the first 8 h after the oral administration, the normal-density gas sensor capsules were identified to be in the pigs’ stomach.

For the measurements using normal-density gas sensor capsules under the thermoneutral condition, the produced CO_2_ concentrations corresponding to the first and second feeding were averaged at ~20% and ~7%, for the control and ~12% and ~3% for the cinnamon-treated pigs, respectively. These numbers were all within the 1–2% variance range compared to those obtained using high-density gastric gas profilers. For the heat-stress condition, the produced CO_2_ concentrations were slightly lower but not in contradiction to those observed using the high-density gas profilers.

## Discussion

The purposes of this pilot study are threefold: to gain insights into the physiological alterations that occur under heat-stress conditions; to better define the effects of cinnamon on those physiological changes; and more importantly, to determine the contribution of the new information from continuous monitoring of CO_2_ concentrations and temperature in the stomach to understanding the physiological processes occurring. The gastric CO_2_ gas measurements are achieved *via* a modified version of the gas sensor capsule that, by virtue of changing its density, is immobilised in the pigs’ stomach during the experiment. Swine are chosen as test subjects due to the similarity of their gastrointestinal tract to that of humans, and to the fact that swine are very susceptible to heat-stress that can potentially make the observations more clear^28^,^38^,^39^. Because of their non-functional sweat glands, pigs rely on panting (hyperventilation) as the primary evaporative heat loss mechanism when under heat-stress, which significantly increases respiration rate but causes further redistribution of the gas content in the blood stream and possibly the gastrointestinal tract^40^. In the present study, observations are limited to the stomach. As the gut is made of different organs that are diverse in their functionalities and physiochemical environments, the investigation of individual organs is essential. The stomach as the area in which significant digestive activities are chemical and biochemical is the first important organ that can be potentially influenced by the ameliorating effect of cinnamon. The stomach is highly sensitive to heat-stress as the health of gastric mucosa is closely related to the gastric mucosal blood flow^41^, and thus redistribution of blood flow driven by heat-stress may potentiate gastric injury *via*, for example, ulcerogenic agents^42^.

Interpretation of the physiological changes that occurred gives insights into the possible therapeutic benefits of diet supplementation. The trend of the changes in CO_2_ defined by the gastric gas profiler is similar to that obtained from measurements in a respiration chamber^43^,^44^, indicating that the gastric CO_2_ production is mainly attributable to the chemical reactions in the acidic environment of the stomach^45^. This is also likely to be accompanied by products of aerobic metabolism of amino acids obtained from protein digestion, which are then chemically decarboxylated into CO_2_ gas^46^. The concomitant rise in gastric temperature is likely to be attributed to metabolic heat generation from the chemical reaction as a result of the protein digestion^47^. The marked reduction in CO_2_ concentration occurring after the second feeding of the day in the thermoneutral cinnamon-treated subject could possibly be related to a chemical reaction of cinnamon in the stomach.

It has been extensively reported in studies both *in vivo* and *in vitro* that cinnamon in its extracted form inhibits gastric acid and pepsin secretion^33^,^48^. In the present study, this is confirmed by the rise in gastric pH and the reduction of pepsin seen in the Raman spectrum. Such a reduction of gastric acid and pepsin secretion eventually leads to a lowered gastric CO_2_ and metabolic heat production, because gastric CO_2_ is mainly produced from the chemical reaction of food in an acidic environment together with the decarboxylation of amino acids upon protein digestion^45^,^46^. These are all related to the concentrations of both gastric acid and pepsin.

During heat-stress, the CO_2_ gas profile looks more like that seen with cinnamon treatment under thermoneutral conditions for the same reason that gastric acid and pepsin secretion falls. Gastric CO_2_ may also be influenced by hyperventilation and subsequent increases CO_2_ loss from the lungs and greater uptake of gastric CO_2_ by the circulation^40^. The plateauing of the skin temperature in the early afternoon is presumably related to the dominant effect of evaporative heat loss by hyperventilation.

The observation that dietary supplementation with cinnamon appeared to reduce gastric CO_2_ production under heat-stress conditions is less easily explained. The increase in gastric acid and pepsin secretion (and presumably metabolic production of CO_2_) relative to those in the heat-stress control pig and relatively reduced respiratory rate associated with heat-stress will tend to increase gastric CO_2_ content. However, cinnamon increases gastric mucosal blood flow^32^,^49^, and this may permit greater CO_2_ exchange. This is supported by the attenuated rise in gastric temperature that might reflect enhanced radiant heat loss from the mucosa. Skin blood flow might also increase with additional radiant heat loss as suggested by the reduced skin temperature in the cinnamon-treated heat-stress pig. Interestingly, rectal temperature is not reduced and this may indicate rectal mucosal blood flow to be unaffected by cinnamon, an untested but feasible hypothesis.

Hence, this pilot study has demonstrated the potential utility of the real-time gastric gas profiler to enhance understanding of the physiological processes that occur in a model of a gastric disorder and of the mechanisms by which a potential therapeutic agent acts. Thus, for the heat-stress effect, the gastric CO_2_ level is found to be suppressed due to the combination of reduction of gastric acid and pepsin secretion as well as hyperventilation, providing a novel marker of the physiological disturbances that enhances information from more traditional physiological and biochemical measurements. The addition of cinnamon into the diet inhibits both the gastric acid and pepsin secretion, resulting in a significant decline in the gastric CO_2_ gas level and alters CO_2_ gas pattern. When under heat-stress, partial amelioration of disorders signified by physiological indices is observed. The CO_2_ gas pattern corresponding to the second feeding of the day is greatly altered as the gastric CO_2_ gas is rapidly depleted during that period, which can be ascribed to the possible enhanced gastric mucosal blood flow in the presence of cinnamon. In addition, the relatively normal gastric acid and pepsin secretion may reflect the potential antioxidant activity of cinnamon against the intestinal mucosal injury induced by tissue hypoxia^32^,^50^. Altogether, it is suggested that cinnamon may play an important role in gastric cytoprotection and maintenance of homeostasis in gastric secretion and digestive functionalities.

The strong similarity and repeatability shown when comparing the gastric gas profiles obtained from the high-density profilers and normal-density gas sensor capsules indicates that the profiles obtained using the transient non-invasive capsules are precise. This suggests that this technology can be reliably used for the interpretation of the gastric gas profiles.

In conclusion, this gastric gas profiler has provided novel, precise and repeatable real-time assessment of gastrointestinal physiological processes that enhance our understanding of a representative gastric disorder induced by heat-stress and the effect of a cinnamon supplement as a dietary remedial model. Since such emerging gas sensor capsule technology can readily be designed to target other parts of the gastrointestinal tract and/or other gas molecules, it presents as a novel medical tool that is capable of providing unique information (particularly *via* the gas level, pattern and production kinetics) about disorders of the gastrointestinal tract. Such information may permit new insights that will underpin future research into pathogenesis of gastrointestinal disorders, dietary and drug effects, and clinical diagnostic applications.

## Methods

### Ethics Statement

All experiments involving pigs were undertaken in accordance with the approved guidelines outlined and approved by the University of Melbourne Faculty of Veterinary and Agricultural Sciences Animal Ethics Committee. Ethics Document No. 1513462, approved on the 5th of June 2015.

### Feeding and environmental manipulation

#### Main tests

Four female crossbred pigs (averaged at ~40 kg) were kept at four different conditions. They were fed a commercial ration (18% crude protein, 13.8 MJ digestible energy/kg) supplemented with either 0 or 1.5% *wt*/*wt* cinnamon (*cinnamomum verum*) and housed under either thermoneutral (20 °C) or heat-stress conditions (35 °C from 09:00–17:00 and 28 °C for the reminder of each 24 h period). Swine were acclimatised to diets for 14 days pre-trial and fed twice a day at around 09:00 and 15:00 over the 2-day experiments. Access to feed and water was provided by troughs and nipple drinkers. Heat and humidity were monitored by using temperature and humidity Dataloggers^®^ placed in the thermally controlled rooms.

#### Repeatability tests

The repeatability of gastric gas profiles were assessed using a group of 12 pigs, which included 4 pigs from the main tests that were administrated with high-density gastric gas profilers and 8 additional pigs that were administrated with normal-density gas sensor capsules. The additional 8 pigs were divided evenly for four different feeding and environmental conditions similar to those in the main tests. This resulted in a total of three pigs for each condition (N = 3).

### Fabrication and deployment of the gastric gas profiler

**Figure 1:**
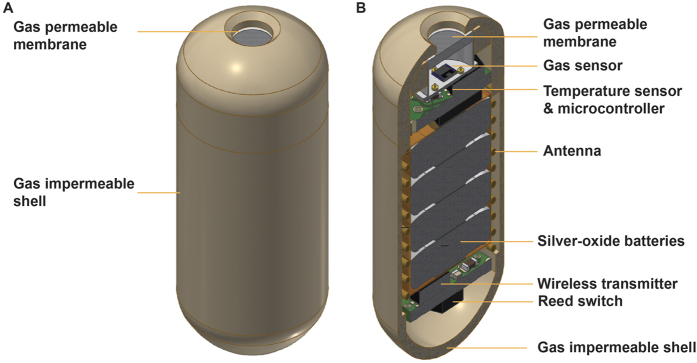
Schematic of a gastric gas profiler (a modified version of gas sensor capsule with higher density): (**A**) whole capsule; (**B**) cross sectional view.

**Figure 2:**
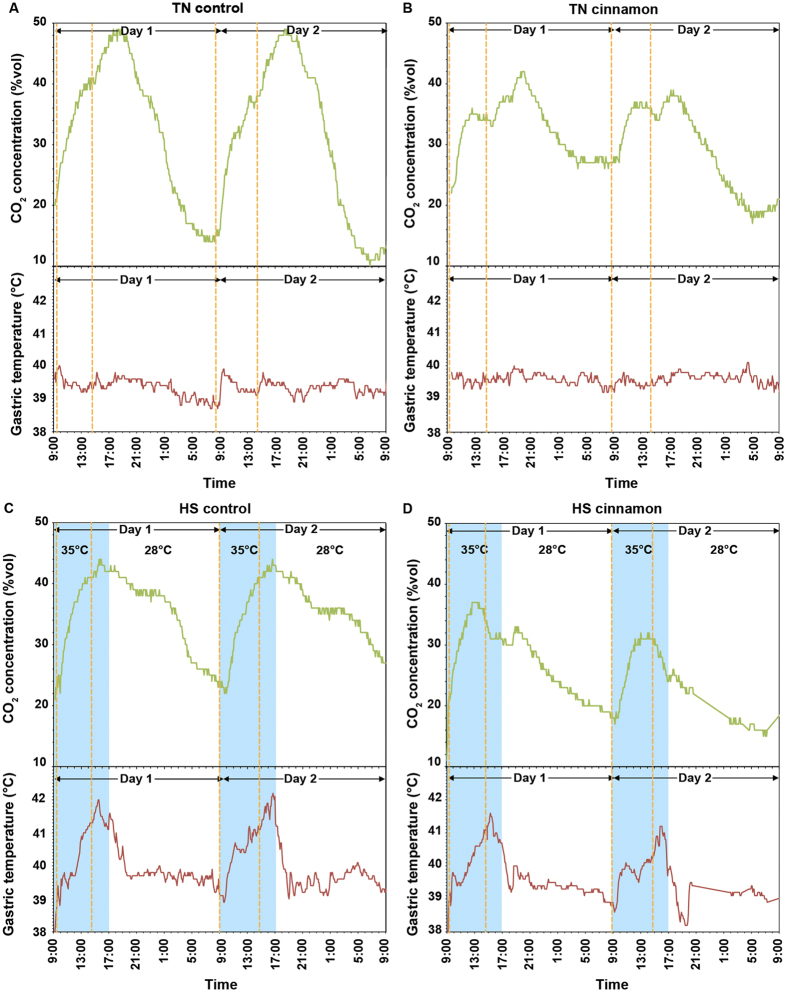
Gastric CO_2_ gas and temperature profiles of: (**A**) thermoneutral (TN) control pig; (**B**) thermoneutral cinnamon-treated pig; (**C**) heat-stress (HS) control pig; and (**D**) heat-stress cinnamon-treated pig. The profiles are obtained using high-density gastric gas profilers. The dotted orange lines indicate the pig feeding times.

**Figure 3:**
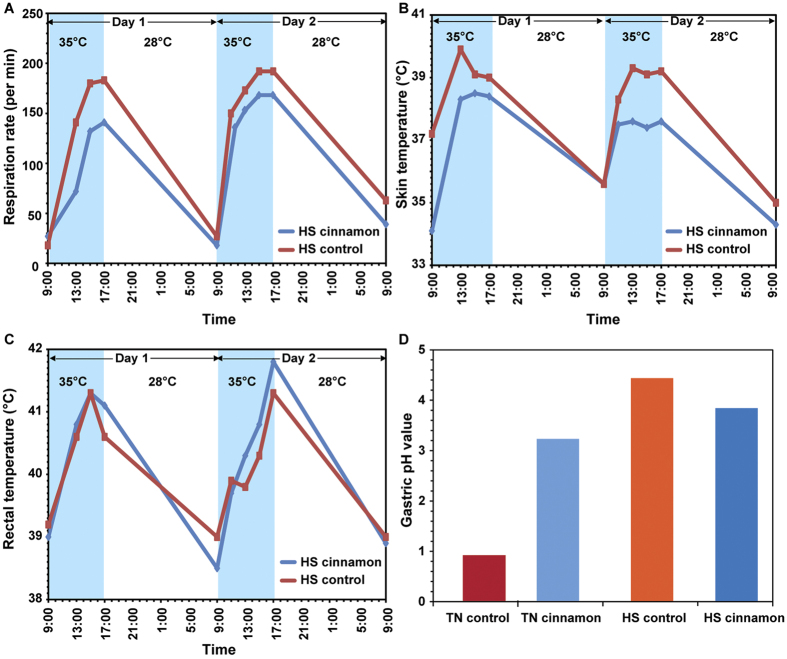
Comparisons of (**A**) respiration rate, (**B**) skin temperature and (**C**) rectal temperature between controlled and cinnamon-treated pigs under heat-stress (HS). (**D**) Gastric pH values of pigs under thermoneutral (TN) and heat-stress conditions.

**Figure 4:**
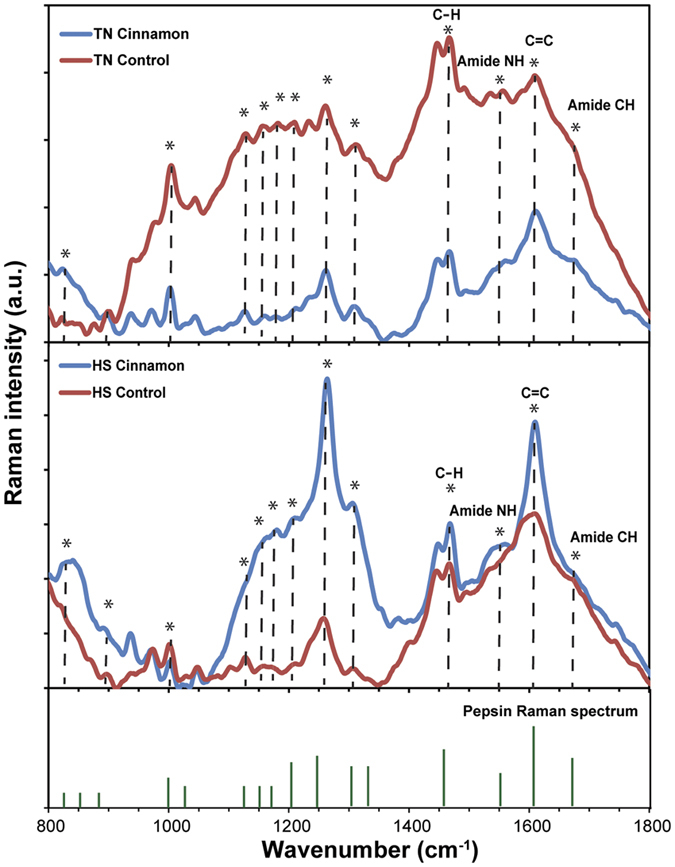
Raman spectra of gastric digesta of dissected pigs under both thermoneutral (TN) and heat-stress (HS) conditions. Annotation *indicates the pepsin Raman signature.

**Figure 5:**
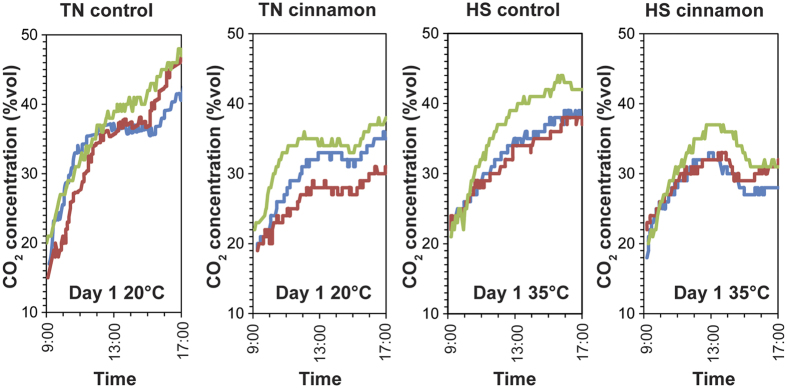
Comparison of gastric CO_2_ gas profiles obtained by using the normal-density gas sensor capsules (blue and red lines) and the high-density gastric gas profilers (green line) in the first 8 h of the experiment for the thermoneutral controlled (TN control), thermoneutral cinnamon-treated (TN cinnamon), heat-stress controlled (HS control) and heat-stress cinnamon-treated (HS cinnamon) subjects.

The design of the gastric gas profiler was similar to our previously implemented gas sensor capsules^26^, with the difference that its density was increased to promote its permanent immobilisation in the pigs’ stomach during the experiment. The high-density gastric gas profiler consisted of gas and temperature sensors, a gas-permeable membrane, micro-electronic circuits, silver-oxide batteries, a reed switch and telecommunication components (including wireless transmitter and antenna), all sealed within a gas impermeable shell (Fig. 1). The gas sensor was based on the thermal conductivity calorimetric technology that has been shown to be sensitive to CO_2_ gas (the selectivity of the gas sensor was presented in Supplementary Information Note 1). As demonstrated in a post-mortem pig study, CO_2_ was the only major gas produced in the stomach in response to a normal diet^51^. The gas profiler was a capsule-shaped device of 2.4 cm length and 1 cm diameter. The resolution of the CO_2_ gas and temperature sensors was 1%vol and 0.2 °C, respectively. The evaluation of the accuracy of the temperature sensor was presented in Supplementary Information Note 2. Gas and temperature profiles were wirelessly transmitted to an external handheld device that allowed for data storage and analysis. The devices were administered by gavaging into the pigs’ stomach and data was acquired at 5 min intervals.

### Physiological measurements

Respiration rate, skin and rectal temperatures were measured at around 09:00, 11:00, 13:00, 15:00 and 17:00 daily. Respiration rate was recorded by observing flank movements and counting the number of breaths per minute. Skin temperature was measured using an infrared thermometer at the skin surface between the leg and torso while rectal temperature was measured using a handheld digital thermometer.

### Blood measurements

Before the completion of the experiment, 1 mL of blood was collected from the ear vein of each pig. The samples were then immediately assayed for acid-base indices, including pH, pCO_2_ (plasma CO_2_) and cHCO_3_^−^ (concentration of bicarbonate in plasma), using an automated blood gas analyser (Epoc^®^, Alere).

### Measurement of the gastric digesta

At the completion of the experiment, pigs were euthanized and dissected. During dissection, the gastric digesta were collected and pH values were immediately measured using a calibrated pH meter (Accumet, AB15). Measurements were repeated five times for each sample and the average calculated.

After pH measurements, gastric digesta were mixed with a 5% *wt*/*wt* solution of silver nanoparticles (Sigma Aldrich, average particle size < 150 nm) at ratio of 1:1. The silver nanoparticles were used for amplifying Raman spectroscopic signals of chemicals presented in the digesta^52^. Subsequently, the mixture was cyclically bath-sonicated for 15 min (4 min sonication/1 min incubation at rest, repeated three times). 40 μL of the mixture was then drop-cast onto clean silicon substrates at 60 °C for Raman spectroscopic measurements (Craic 20–30 microspectrophotometer). An excitation wavelength of 785 nm at 1 mW power was used in the experiment (accuracy of ±4 cm^−1^). The final intensity of the Raman spectrum of each sample was average of four repeated Raman scans with an exposure time of 30 s each. Background luminescent offset, noise removal and baseline adjustment of the Raman spectra were conducted using a Matlab^®^ 10 software package.

## Additional Information

**How to cite this article**: Ou, J. Z. *et al.* Potential of *in vivo* real-time gastric gas profiling: a pilot evaluation of heat-stress and modulating dietary cinnamon effect in an animal model. *Sci. Rep.* **6**, 33387; doi: 10.1038/srep33387 (2016).

## Supplementary Material

Supplementary Information

## Figures and Tables

**Table 1:** Blood parameters of pigs under both thermoneutral and heat-stress conditions.

Parameters	Thermoneutral		Heat-stress	
	Control	Cinnamon	Control	Cinnamon
pH	7.41	7.40	7.42	7.42
pCO_2_ (mm Hg)	55.5	53.5	50.1	50.2
cHCO_3_^−^ (mmoL/L)	35.5	32.9	33.3	32.7
