# Supplementary material for: Potential of in vivo real-time gastric gas profiling: a pilot evaluation of heat-stress and modulating dietary cinnamon effect in an animal model
Source: Sci Rep. 2016 Sep 16;6:33387. doi: 10.1038/srep33387 (PMC5025890; doi:10.1038/srep33387)
Supplement: Supplementary Information [file srep33387-s1.pdf]

Supplementary Information

Potential of *in vivo* real-time gastric gas profiling: a pilot evaluation of heat-stress and modulating dietary cinnamon effect in an animal model

Jian Zhen Ou[1], Jeremy J Cottrell[2], Nam Ha[1], Naresh Pillai[1], Chu K Yao[3], Kyle J Berean[1], Stephanie A Ward[4], Danilla Grando[5], Jane G Muir[3], Christopher J. Harrison[1], Udani Wijesiriwardana[2], Frank R Dunshea[2*], Peter R Gibson[3*] and Kourosh Kalantar-zadeh[1*]

[1]School of Engineering, RMIT University, Melbourne, Australia

[2]Faculty of Veterinary and Agricultural Sciences, The University of Melbourne, Parkville, Australia

[3]Department of Gastroenterology, The Alfred Hospital, Monash University, Melbourne, Australia

[4]Monash Ageing Research Centre, Monash University, Melbourne, Australia

[5]School of Science, RMIT University, Bundoora, Australia

Supplementary Information Note 1: Selectivity of the gas sensor

The selectivity of the gas sensor is evaluated under the exposure to different gases listed in the introduction, including CO_2, H_2, CH_4, H_2S and NO. According to the post-mortem pig study by Jensen and Jorgensen [1], the concentration of CO_2 as the major gas in the stomach varies from ~12 to ~50% in response to a normal diet, while there is a small amount of H_2 ranged between ~0.5% and ~1%. In addition, no CH_4 gas is detected in the stomach and there is no known literature on the presence of other gases such as H_2S and NO in the stomach of pigs. Therefore, for the selectivity assessments for our sensor, the concentrations for CO_2 and H_2 are chosen similar to those presented by Jensen and Jorgensen. For CH_4, H_2S and NO, the concentrations are selected as 6%, 56 ppm and 10 ppm, respectively. These concentrations reach either the combustion lower limit (CH_4) or the immediately dangerous to life or health (IDLH) values (H_2S and NO). The influence of humidity on the gas sensor response is also investigated. Two relative humidity (R. H.) levels are chosen, in which one is at ~30% that represents the ambient condition and the other is ~100% that reflects the actual gut environment closely.

From Figure S1, it is observed that only CO_2 gas causes the increase of sensor output voltage while the responses for humidity, H_2 and CH_4 are opposite. There is no obvious response toward both H_2S and NO. In addition, the response magnitude for CO_2 is much larger (up to 0.06 V) compared to H_2 (up to 0.015 V), CH_4 (up to 0.01 V) and humidity (up to 0.005 V), which indicates high selectivity of this gas sensor in the pig stomach environment. Therefore, it can be concluded that the sensor responses shown in Figure 2 are the result of the change of gastric CO_2 concentration in response to diet and stress.

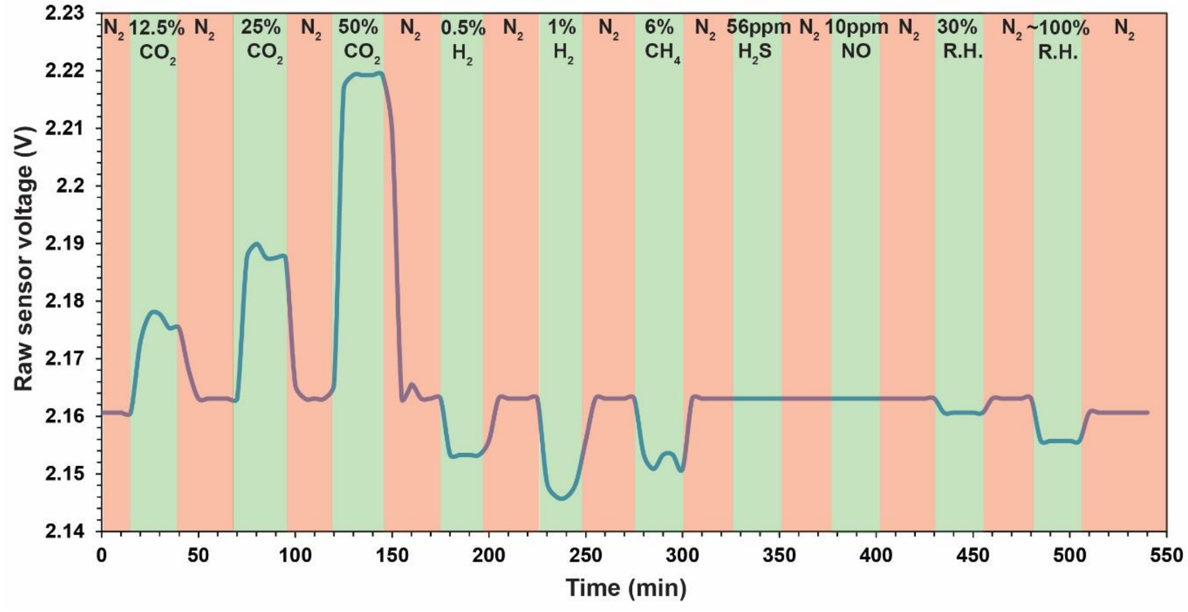

Figure S1. Selectivity measurement of the gas sensor of the gastric gas profiler in the presence of CO_2 (12.5-50%), H_2 (0.5-1%), CH_4 (6%), H_2S (56 ppm), NO (10 ppm) and humidity (30% and ~100% R. H.) in dry N_2 balance.

Supplementary Information Note 2: Accuracy of the temperature sensor

The accuracy of the temperature sensor is investigated by placing the gastric gas profiler in an environmental chamber at a temperature range between 38°C and 42°C, which simulates the gastric temperature variation in response to diet and stress (Figure 2). By comparing the sensor digital reading with the actual chamber temperature, the temperature sensor has a high accuracy with the noise level of only up to 0.2°C as shown in Figure S2, which is similar to that of the chamber temperature controller (0.2°C accuracy). The rise of temperature corresponding to feeding without cinnamon in the thermoneutral condition is between 0.6°C and 0.8°C (Figure 2A) which is well above the noise level of the temperature sensor, confirming that the heat production is due to metabolic and chemical activities. In comparison, the temperature variation corresponding to the feeding with cinnamon is between ~0.2 and ~0.4°C (Figure 2B), which is just above the noise floor. This means that the noise analysis for the temperature profile confirms the conclusion that the addition of cinnamon in the diet possibly reduces the heat production due to the inhibition of both the gastric acid and pepsin secretion.

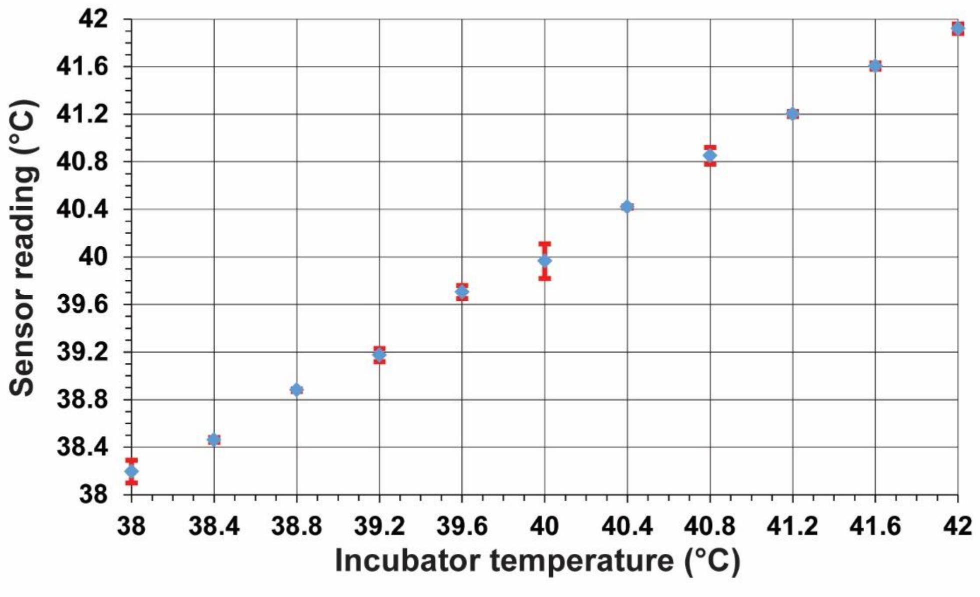

Figure S2. The digital reading of the temperature sensor in the gastric gas profiler *vs* the actual environmental chamber temperature.

Reference

1 Jensen, B. B. & Jorgensen, H. Effect of dietary fiber on microbial activity and microbial gas production in various regions of the gastrointestinal tract of pigs. *Appl. Environ. Microbiol.* **60**, 1897-1904, (1994).
